# α-Synuclein impairs ferritinophagy in the retinal pigment epithelium: Implications for retinal iron dyshomeostasis in Parkinson’s disease

**DOI:** 10.1038/s41598-017-12862-x

**Published:** 2017-10-09

**Authors:** Shounak Baksi, Neena Singh

**Affiliations:** Department of Pathology, School of Medicine, Case Western Reserve University, Cleveland, Ohio 44106 USA

## Abstract

Retinal degeneration is prominent in Parkinson’s disease (PD), a neuromotor disorder associated with aggregation of α-synuclein (α-syn) in the substantia-nigra (SN). Although α-syn is expressed in the neuroretina, absence of prominent aggregates suggests altered function as the likely cause of retinal pathology. We demonstrate that α-syn impairs ferritinophagy, resulting in the accumulation of iron-rich ferritin in the outer retina *in*-*vivo* and retinal-pigment-epithelial (RPE) cells *in-vitro*. Over-expression of Rab1a restores ferritinophagy, suggesting that α-syn impairs lysosomal function by disrupting the trafficking of lysosomal hydrolases. Surprisingly, upregulation of ferritin in RPE cells by exogenous iron *in*-*vitro* stimulated the release of ferritin and α-syn in exosomes, suggesting that iron overload due to impaired ferritinophagy or other cause(s) is likely to initiate prion-like spread of α-syn and ferritin, creating retinal iron dyshomeostasis and associated cytotoxicity. Since over-expression of α-syn is a known cause of PD, these results explain the likely cause of PD-associated retinal degeneration.

## Introduction

Parkinson’s disease (PD) is a neurodegenerative condition caused by the death of dopaminergic neurons in the substantia nigra (SN)^1^. Apart from the typical neuro-motor symptoms resulting from the lack of dopamine (DA), visual symptoms ranging from impairment of visual acuity, contrast sensitivity, color vision, and motion perception are a significant cause of PD-associated morbidity, and are hypothesized to arise from degeneration of the retina^2–4^. The underlying cause of retinal pathology, however, has remained unclear. Efforts at identifying aggregates of α-synuclein (α-syn) in the retina, the principal pathogenic feature diagnostic of sporadic and certain familial forms of PD, have been unsuccessful, leaving the matter unsettled^5,6^. However, the option of using retinal thickness as a potential diagnostic test for PD requires reevaluation of this question that has received little attention because of its relatively innocuous nature.

How might PD pathology involve the retina? The pathogenesis of PD is complex. Most cases of PD are sporadic in nature. Only 10% are familial in origin. However, as for other neurodegenerative conditions of uncertain etiology, familial forms of PD have provided important information on the pathogenesis of sporadic forms that are difficult to model. Till date, mutations in 18 specific chromosomal regions termed *PARK* have been linked to PD. Point mutations or gene duplications/triplications in specific gene loci increase the risk for PD or result in autosomal dominant or recessive PD^7^. Of these, *SNCA* (*PARK1*) encoding α-syn has attracted significant attention because duplications/triplications in *SNCA* result in autosomal dominant PD, and the age of onset, clinical symptoms, and severity of phenotype correlate with gene dosage^8^. α-Syn is degraded in lysosomes, and also inhibits lysosomal activity by disrupting the transport of lysosomal hydrolases by interacting with the Rab family of proteins, forming a positive feed-back loop that results in the accumulation of α-syn^9,10^. It is interesting to note that five other genes linked unambiguously to PD, i.e. leucine-rich repeat kinase 2 or *LRRK2* (*PARK8*), Parkinson protein 2 E3 Ubiquitin protein ligase or Parkin (*PARK2*), PTEN Induced Putative Kinase 1 or *PINK1* (*PARK6*), parkinsonism-associated deglycase or *DJ-1* (*PARK7*), and ATPase Type 13A2 or *ATP13A2* (*PARK9*) are all involved in protein turnover by the autophagy-lysosomal pathway (ALP)^7^, underscoring the significance of this pathway in the pathogenesis of PD.

An additional feature complicating sporadic and familial PD is iron dyshomeostasis in the SN^11^. Both neurons and microglia accumulate iron, and the extent of iron deposition correlates with the severity of symptoms^11–16^. The increase in neuronal iron is mainly attributed to upregulation of the iron uptake protein divalent metal transporter (DMT1) and downregulation of the iron export protein ferroportin^17^. Microglia, on the other hand, are believed to accumulate iron from phagocytosed iron-rich neurons. This phenotype is unusual, and reflects PD-specific dysregulation of the iron homeostatic machinery. Normally, cellular iron homeostasis is maintained by the coordinated expression of iron uptake, storage, and efflux proteins regulated at the transcriptional and translational level by a set of iron regulatory proteins. During iron overload, iron uptake proteins are down-regulated, and the only known iron export protein ferroportin is upregulated^18^. The opposite scenario occurs during iron deficiency. Ferritin, the major iron storage protein in all cells, is of prime importance in maintaining safe levels of intracellular iron by sequestering iron during excess, and releasing stored iron during deficiency^19^. The latter requires efficient delivery of ferritin to the autophagophore by the Nuclear Receptor Coactivator 4 (NCOA4)^20,21^, followed by membrane association of microtubule associated protein 1 Light Chain 3 (LC3) and conversion of cytoplasmic LC3I to phosphatidylethanolamine bound LC3II^22^. The mature autophagosome thus formed merges with the lysosome, where the autophagic load including ferritin is degraded by lysosomal hydrolases^23–25^ and the released iron used to replenish the cellular labile iron pool. The latter process is termed ferritinophagy^20^. Since α-syn disrupts lysosomal activity^10^, it is likely that degradation of ferritin is affected as well, resulting in sequestration of iron in ferritin and a phenotype of functional iron deficiency.

To evaluate whether α-syn influences cellular iron homeostasis by modulating the turnover of ferritin, we used the neuroretina from wild-type (α-syn^+/+^) and α-syn knock-out (α-syn^−/−^) mice and a human retinal pigment epithelial cell line (RPE47) as experimental models. Several factors prompted the use of this model: 1) retinal degeneration is a prominent feature of PD pathology, and occurs relatively early in the clinical course^2,3^, 2) α-syn is expressed in all layers of the neuroretina, including the RPE cell layer^5^, 3) RPE cells, like DA neurons of the SN, synthesize DA and melanin^26,27^, 4) RPE cells possess a highly developed phagocytic apparatus responsible for degrading outer tips of photoreceptor cells, a function necessary for visual function^28^, 5) The photoreceptors are rich in ferritin, placing an immense burden on RPE cells for its turnover and the management of released iron^29^, and finally, 6) the retina is relatively more accessible than neurons of the SN as an experimental model.

Using this approach, we report that α-syn interferes with ferritinophagy, resulting in functional iron deficiency despite the presence of iron-loaded ferritin. Over-expression of Rab1a rescues this phenotype, implicating α-syn-mediated lysosomal dysfunction in PD-associated iron dyshomeostasis. Surprisingly, exposure of RPE cells to excess iron stimulates the release of α-syn and ferritin in exosomes, a novel observation partly explaining the prion-like spread of α-syn in PD brain.

## Results

### α-Syn impairs ferritinophagy in the retina

**Figure 1:**
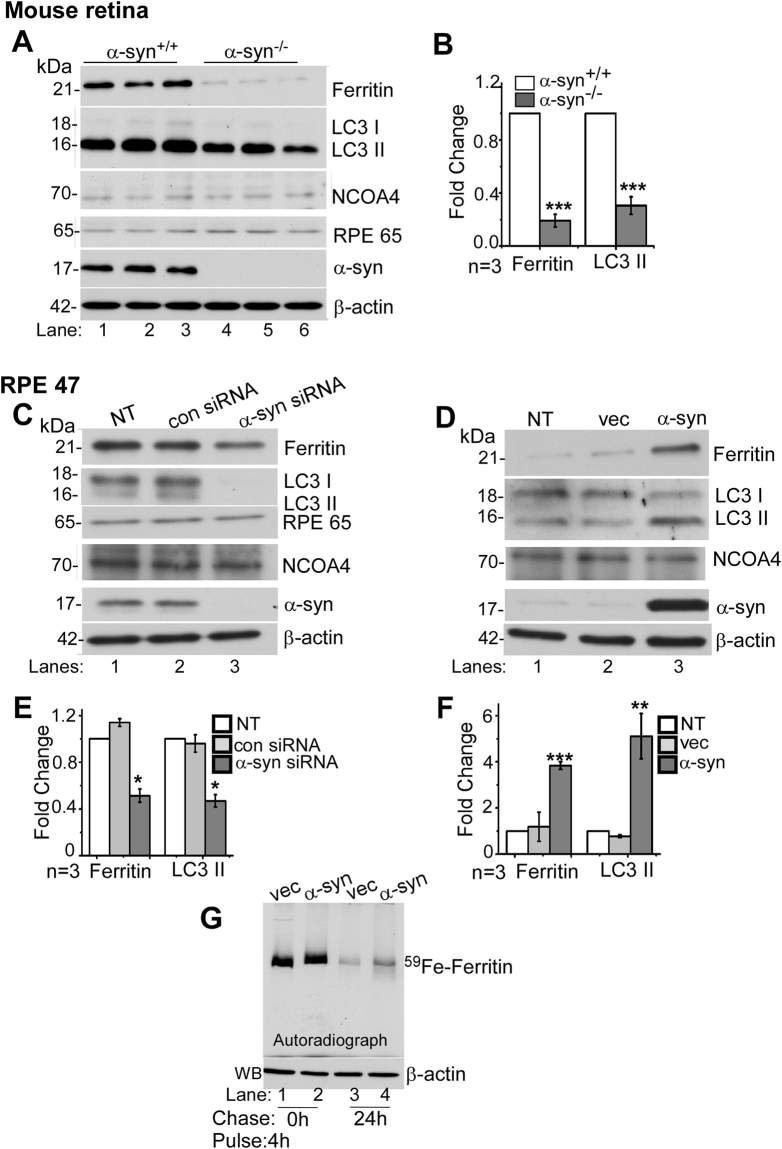
α-Syn inhibits the degradation of ferritin and LC3II: Fig. 1: (**A**) Representative Western blot of retinal lysates from α-syn^+/+^ and α-syn^−/−^ mice (lanes 1–3 vs. 4–6) shows expression of ferritin, LC3, α-syn, RPE65^27^, and NCOA4^20^. (**B**) Quantification by densitometry of ferritin and LC3II in α-syn^−/−^ relative to α-syn^+/+^ samples. (**C**) Lysates of RPE 47 cells transfected with siRNA for α-syn, scrambled siRNA, and non-transfected controls were analyzed by Western blotting. Representative image shows expression of ferritin, LC3, RPE 65, NCOA4, α-syn and β-actin. (**D**) Expression of ferritin, LC3, NCOA4, α-syn and β-actin in non-transfected and RPE 47 cells stably expressing vector or α-syn. (**E**) Quantification of ferritin and LC3II expression by densitometry following knockdown of α-syn. (**F**) Quantification of ferritin and LC3II following over-expression of a-syn. (**G**) ^59^Fe-feriitin in vector and α-syn expressing RPE 47 cells pulsed with ^59^FeCl_3_ for 4 h (lanes 1 & 2) and chased for 0 h and 24 h. Western blotting of same volume of samples as a control for protein loading. n = 3 for all experiments. All values were normalized to β-actin that served as an internal control, and represent mean ± SEM of the indicated *n* (**p < 0.01, **p < 0.01 ***p < 0.001).

To evaluate whether α-syn modulates ferritinophagy, levels of ferritin and LC3II, the latter a marker of autophagy^22^, were assessed in retinal lysates including the RPE cell layer harvested from α-syn^+/+^ and α-syn^−/−^ mice (Fig. 1A). Expression of ferritin was lower in α-syn^−/−^ samples as reported previously (Fig. 1A, lanes 1–3 vs. 4–6; Fig. 1B)^30^. However, levels of LC3II were also reduced in α-syn^−/−^ samples (Fig. 1A, lanes 1–3 vs. 4–6; Fig. 1B), a surprising observation since iron deficiency is expected to increase the turnover of ferritin by activating autophagy^21^. Re-probing for Retinal Pigment Epithelium Specific Protein 65 (RPE65) and α-syn confirmed that the samples were representative of the retina, including the RPE cell layer, from α-syn^+/+^ and α-syn^−/−^ mice (Fig. 1A, lanes 1–6). Re-probing for NCOA4 showed no significant difference between the two mouse lines (Fig. 1A, lanes 1–3 vs. 4–6), making it unlikely that the difference in ferritin levels is due to inefficient delivery to the autophagophore.

To resolve the above dichotomy, further experiments were performed *in vitro* in a RPE cell line that expresses abundant α-syn^5,6,31^. Since autophagic flux and LC3 levels vary with cell cycle^32^, cells were cultured from 12 and 48 h in different experiments before evaluation, keeping the time constant within one experiment. This approach resulted in variable expression of LC3I and LC3II between experiments, but reduced bias due to cell-cycle.

A knock-down of α-syn with RNAi reduced both ferritin and LC3II relative to controls, reproducing the observations with α-syn^−/−^ samples in panel A (Fig. 1C, lane 3 vs. 1 & 2, Fig. 1E). Over-expression of α-syn increased ferritin and LC3II levels (Fig. 1D, lane 3 vs. 1 & 2; Fig. 1F), indicating blockage of ALP^22^. The paradoxical increase in ferritin despite increased phagocytic activity suggested either impaired fusion of autophagosomes with lysosomes, or dysfunction of lysosomal activity downstream from this step. No change in NCOA4 suggested that delivery of ferritin to phagosomes is unaffected by α-syn.

Next, the release of iron from ferritin was monitored in RPE cells, a process that requires degradation of ferritin^23^. Thus, equal number of cells over-expressing α-syn or vector were radiolabeled with equal counts of ^59^Fe-citrate for 4 h, followed by wash-out and chase in normal medium for 24 h. Lysates were fractionated by native gel electrophoresis followed by autoradiography to visualize ^59^Fe-ferritin as in previous reports^33–35^. Protein loading was normalized by fractionating equal volume of lysate samples supplemented with denaturing buffer on SDS-PAGE followed by immunoblotting for β-actin. As expected, a prominent band of ^59^Fe-ferritin was noted in vector and α-syn expressing cells after a pulse of 4 h (Fig. 1G, lanes 1 & 2). Following a chase of 24 h, more than one half-life of ferritin^36^, ^59^Fe-ferritin in α-syn-expressing cells was 2.5-fold higher than vector-expressing controls, indicating impaired degradation and release of iron from ferritin in the presence of α-syn (Fig. 1G, lanes 3 & 4).

The above results suggested that α-syn impairs the degradation and release of iron from ferritin. This is more prominent in cells over-expressing α-syn, but is also noted in the retina of wild-type mice expressing normal levels of α-syn relative to knock-out controls. Further studies were aimed at identifying the mechanism underlying this process.

### α-Syn inhibits autophagosomal/lysosomal activity

**Figure 2:**
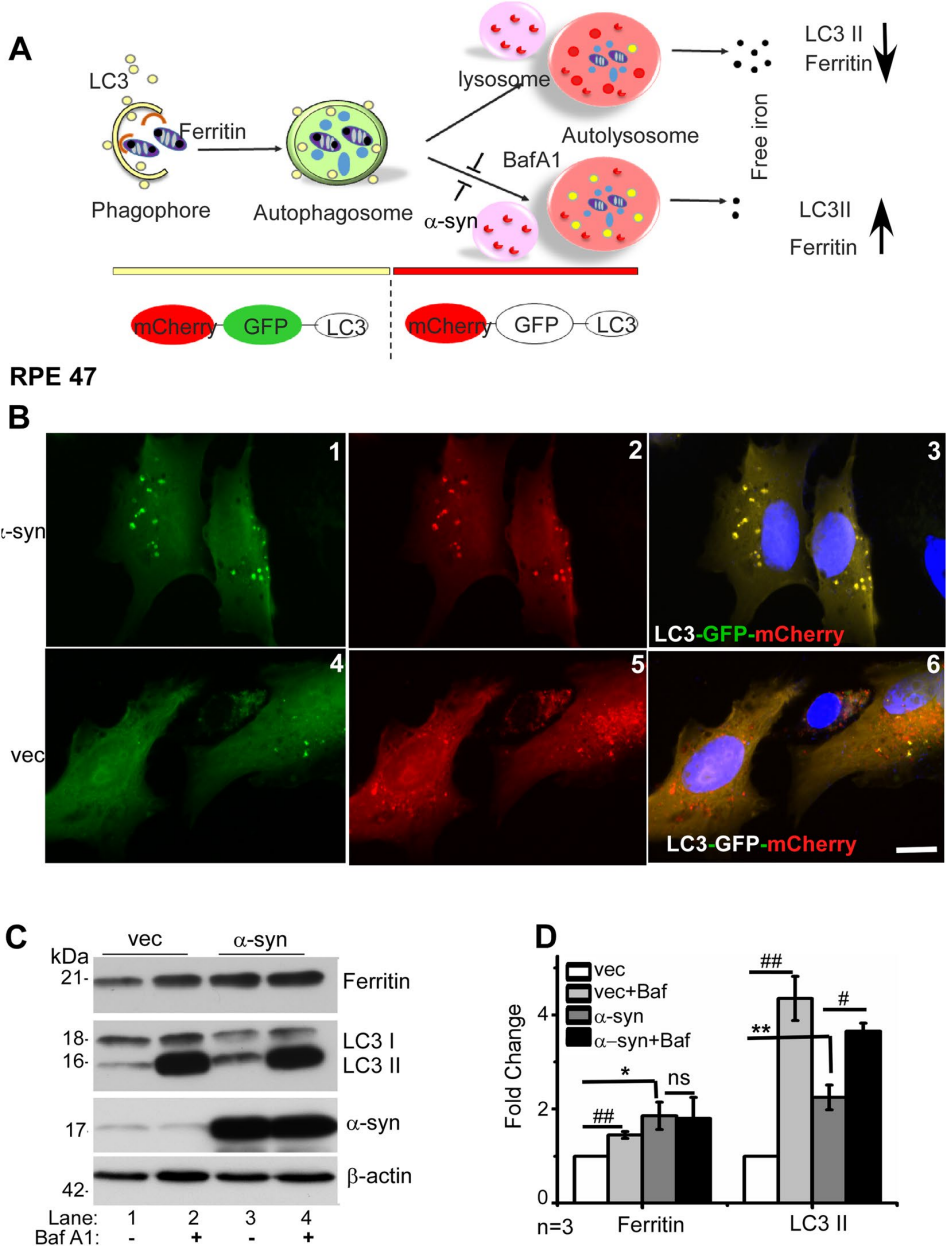
α-Syn impairs lysosomal function in RPE cells: (**A**) When expressed in cells, LC3-GFP-mCherry provides a convenient way to monitor the fusion of LC3 positive autophagosomes with lysosomes. Both GFP and mCherry fluoresce at the neutral pH of autophagosomes, emitting a yellow color. Upon fusion with lysosomes, GFP is quenched due to low pH, while mCherry continues to fluoresce. Efficient fusion of autophagosomes with lysosomes will therefore result in mainly red fluorescence, and the expected degradation of LC3II and ferritin. A block in the fusion of autophagosomes with lysosomes or elevated pH of lysosomes, mimicked pharmacologically by BafA1, will not quench GFP, resulting in yellow fluorescence, and sparing of LC3II and ferritin. (**B**) Representative images of LC3-GFP-mCherry transfected RPE 47 cells stably overexpressing vector or α-syn show a yellow fluorescence in vesicular structures representing autophagosomes, and red fluorescence in lysosomes due to quenching of GFP at low pH. (**C**) Western blot image demonstrating expression of ferritin, LC3, α-syn, and β-actin in RPE cells overexpressing α-syn or vector following treatment with 100 µM BafA1 for 12 h. (**D**) Quantification by densitometry after normalization with β-actin. n = 3. Values represent mean ± SEM of the indicated *n* (*p < 0.05, **p < 0.01, #*p* < 0.05, ##*p* < 0.01). Asterisk (*) and hash (#) signs show comparison of untreated α-syn expressing cells with vector (*) and Baf A1 treated cells with untreated controls (#) respectively.

To evaluate whether α-syn interferes with the fusion of autophagosomes with lysosomes, a widely used technique utilizing the pH-sensitive characteristics of GFP and mCherry tagged to the N-terminus of LC3 plasmid was used (Addgene #22418)^37^. At the physiological pH of phagophores and autophagosomes, both eGFP (green) and mCherry (red) fluoresce, emitting a yellow fluorescence. The green fluorescence of eGFP (acid sensitive) is quenched at the low pH of lysosomal, and only the red fluorescence of mCherry (acid stable) is visible. The ratio of red vs. yellow (green/red) vesicles therefore provides an estimate of the flux of autophagic vesicles to the lysosomal compartment. Bafilomycin A1 (Baf A1), a V-ATPase that inhibits the acidification of endosomes provides a negative control (Fig. 2A).

Thus, eGFP-mCherry-LC3 was expressed in RPE cells overexpressing α-syn and vector controls, and the cells were imaged 48 h following transfection. Surprisingly, all the fluorescent vesicles in α-syn over-expressing cells emitted yellow fluorescence, indicating either lack of fusion of autophagosomes with autolysosomes, or an abnormally high pH in the latter compartment (Fig. 2B, panels 1–3). In vector transfected cells, on the other hand, most of the vesicles fluoresced red, indicating quenching of eGFP due to the low pH of autolysosomes as expected (Fig. 2B, panels 4–6).

To confirm the above observations, vector and α-syn over-expressing cells were treated with Baf A1 for 12 h and the lysates were subjected to immunoblotting. Probing for ferritin revealed significantly higher levels in α-syn over-expressing cells relative to vector controls (Fig. 2C, lanes 1 & 3; Fig. 1D). Exposure to Baf A1 revealed an increase in ferritin in vector transfected cells due to impaired degradation in the lysosomes, but did not show a significant difference in α-syn over-expressing cells probably due to α-syn mediated dysfunction of the lysosomes as observed in Fig. 2B above (Fig. 2C, lanes 1 vs. 2 & 3vs. 4; Fig. 2D). Probing for LC3 showed significantly higher levels in α-syn over-expressing cells relative to vector controls, indicating stimulation of the autophagosomal pathway but impaired degradation in the lysosomal compartment (Fig. 2C, lanes 1 & 3; Fig. 2D). Exposure to Baf A1 increased LC3II levels in both vector and α-syn over-expressing cells (Fig. 2C, lane 1 vs. 2 & 3 vs. 4; Fig. 2D), mimicking the effect of α-syn over-expression on the autophagosome/lysosome pathway.

Together, these results confirm that ferritin is degraded by the ALP in RPE cells, and α-syn inhibits this pathway downstream of the autophagosome formation, possibly in the lysosomes.

### Ferritin accumulates in autophagosomes/lysosomes of α-syn over-expressing cells

**Figure 3:**
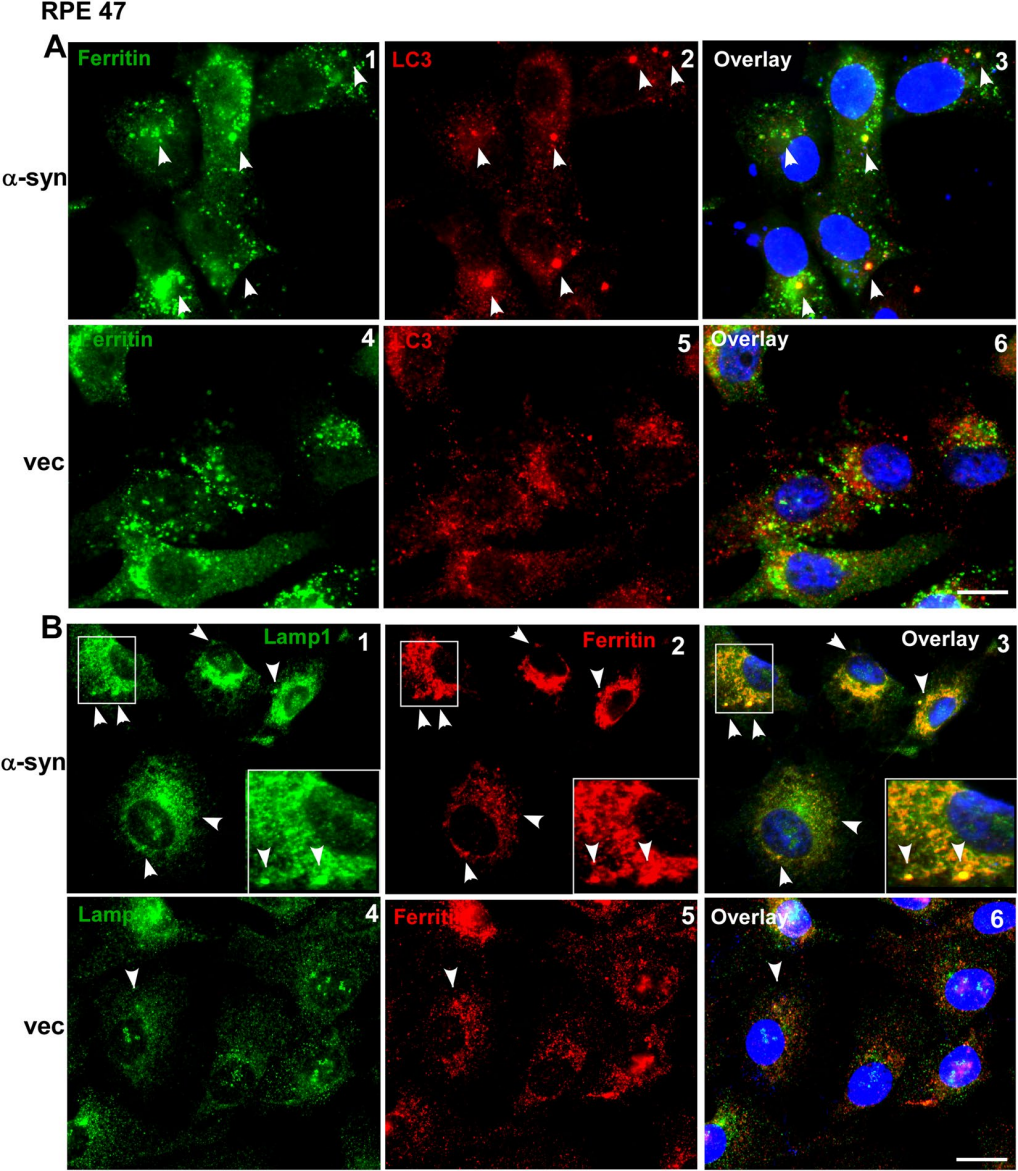
Ferritin co-localizes with LC3 and Lamp1 in α-syn over-expressing cells: (**A**) Co-immunostaining of α-syn and vector-expressing cells for ferritin (green) and LC3 (red) in α-syn and vector expressing cells. (**B**) Co-immunostaining for ferritin (red) and Lamp1 (green) in the same cells. Scale bar 10 µm.

To ascertain whether ferritin accumulates in the autophagosomes or lysosomes of cells over-expressing α-syn, subcellular localization of ferritin was evaluated by immunolocalization studies. Thus, cells over-expressing α-syn and vector controls were co-immunostained with ferritin and the autophagosome marker LC3 (Fig. 3A), or the lysosomal marker LAMP1 (Fig. 3B). Significant co-localization of ferritin with LC3 and LAMP1 was observed in α-syn over-expressing cells relative to vector controls where most of the ferritin was cytosolic as expected (Fig. 3A & B, panels 1–3 vs. 4–6).

These observations suggest that ferritin follows the autophagic pathway for its delivery and ultimate degradation in lysosomes to release the bound iron^19–21,24^. Almost complete co-localization of ferritin with lysosomes in α-syn over-expressing cells suggests that ferritin is delivered to lysosome, but its degradation is impaired by α-syn.

### α-Syn impairs ferritinophagy following light-induced photoreceptor damage

**Figure 4:**
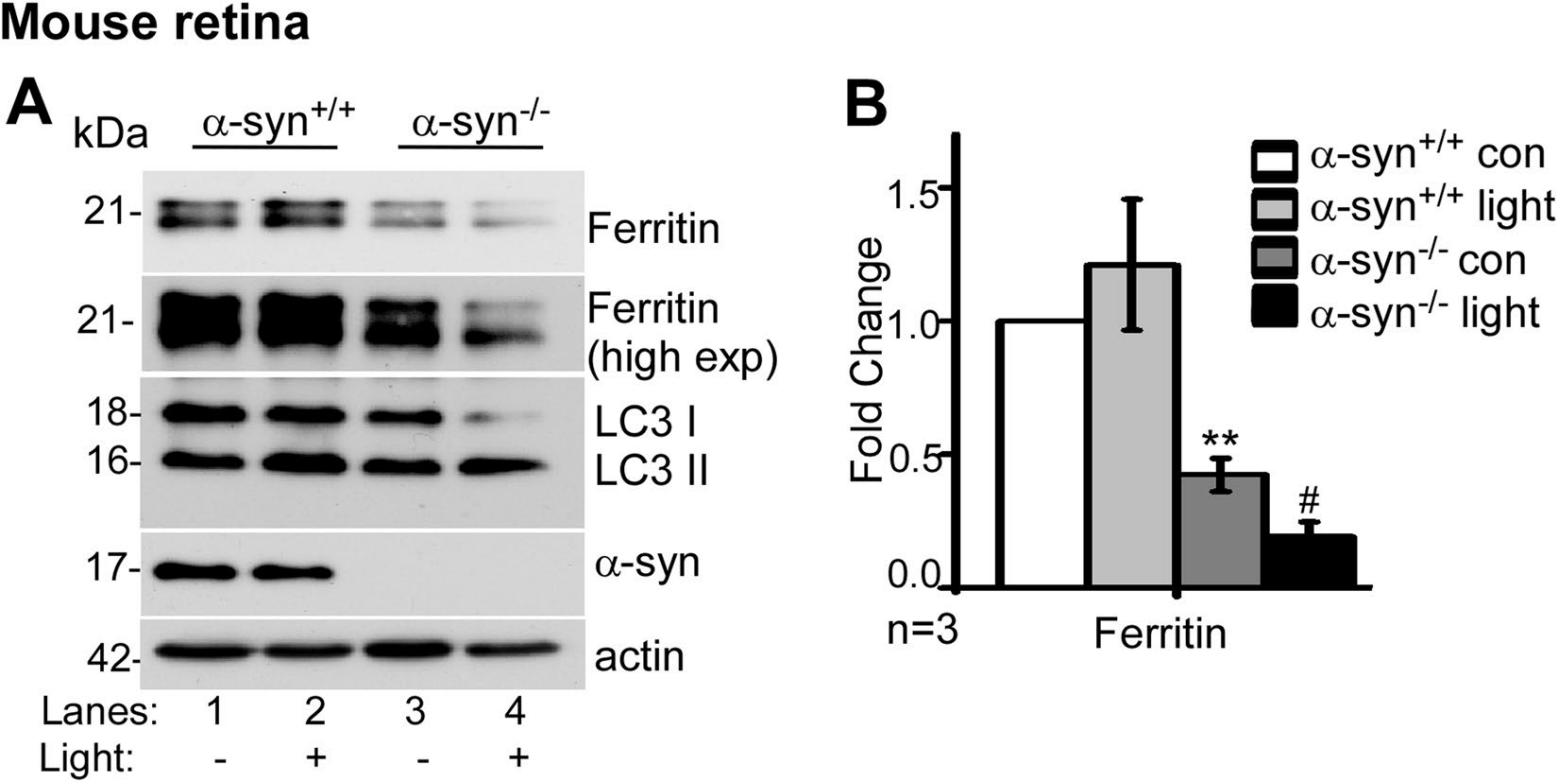
α-Syn impairs ferritinophagy following light-induced photoreceptor damage: (**A**) Western blot image demonstrating expression of ferritin, LC3, α-syn and β-actin in retinal lysates of light-exposed and control α-syn^+/+^ and α-syn^−/−^ mice. (**B**) Quantification by densitometry after normalization with β-actin. n = 3, Values are mean ± SEM of the indicated *n*. **p < 0.01, #*p* < 0.05). Asterisk (*) indicates comparison of untreated α-syn^−/−^ with untreated α-syn^+/+^ samples, and hash (#) indicates comparison of light-exposed α-syn^−/−^ with untreated α-syn^−/−^ controls.

Phagocytosis and turnover of photoreceptors outer segment (POS) by RPE is important for retinal health and normal vision. This process releases a significant amount of iron which is stored in ferritin in RPE cells. Eventually ferritin is degraded by lysosomes, and the released iron is re-utilized for metabolic purposes or exported from the retina^27,29^. Exposure to high intensity light damages the photoreceptors, thereby inducing autophagy^38^ an, and likely, ferritinophagy. To evaluate whether α-syn interferes with this process, α-syn^+/+^ and α-syn^−/−^ mice were exposed to 10,000 Lux light for 30 min, followed by recovery in the dark for 24 h. The eyes were dissected and the retina harvested and subjected to Western blotting (Fig. 4).

Probing for ferritin revealed significantly less expression in α-syn^−/−^ samples relative to α-syn^+/+^ controls as noted in Fig. 1A (Fig. 4A, lanes 1 & 2 vs. 3 & 4; Fig. 4B). Exposure to light caused minimal change in ferritin in α-syn^+/+^ samples relative to untreated controls (Fig. 4A, lanes 1 & 2; Fig. 4B). In α-syn^−/−^ samples, on the other hand, light damage resulted in a significant decrease in ferritin related to untreated controls (Fig. 4A, lanes 3 & 4; Fig. 4B). Probing for LC3II revealed adequate stimulation of autophagy in the retina of both α-syn^+/+^ and α-syn^−/−^ mice following light exposure.

The above results confirm that degradation of ferritin is significantly more in α-syn^−/−^ relative to α-syn^+/+^ samples following activation of ALP by light-induced damage.

### α-Syn-mediated disruption of ferritinophagy is rescued by Rab1a

α-Syn is known to mediate vesicular trafficking by interacting with members of the Rab family of adaptor proteins^39,40^. A recent report demonstrated impaired trafficking of lysosomal hydrolases in α-syn overexpressing neurons, and rescue of this phenotype by Rab1a^10^.

**Figure 5:**
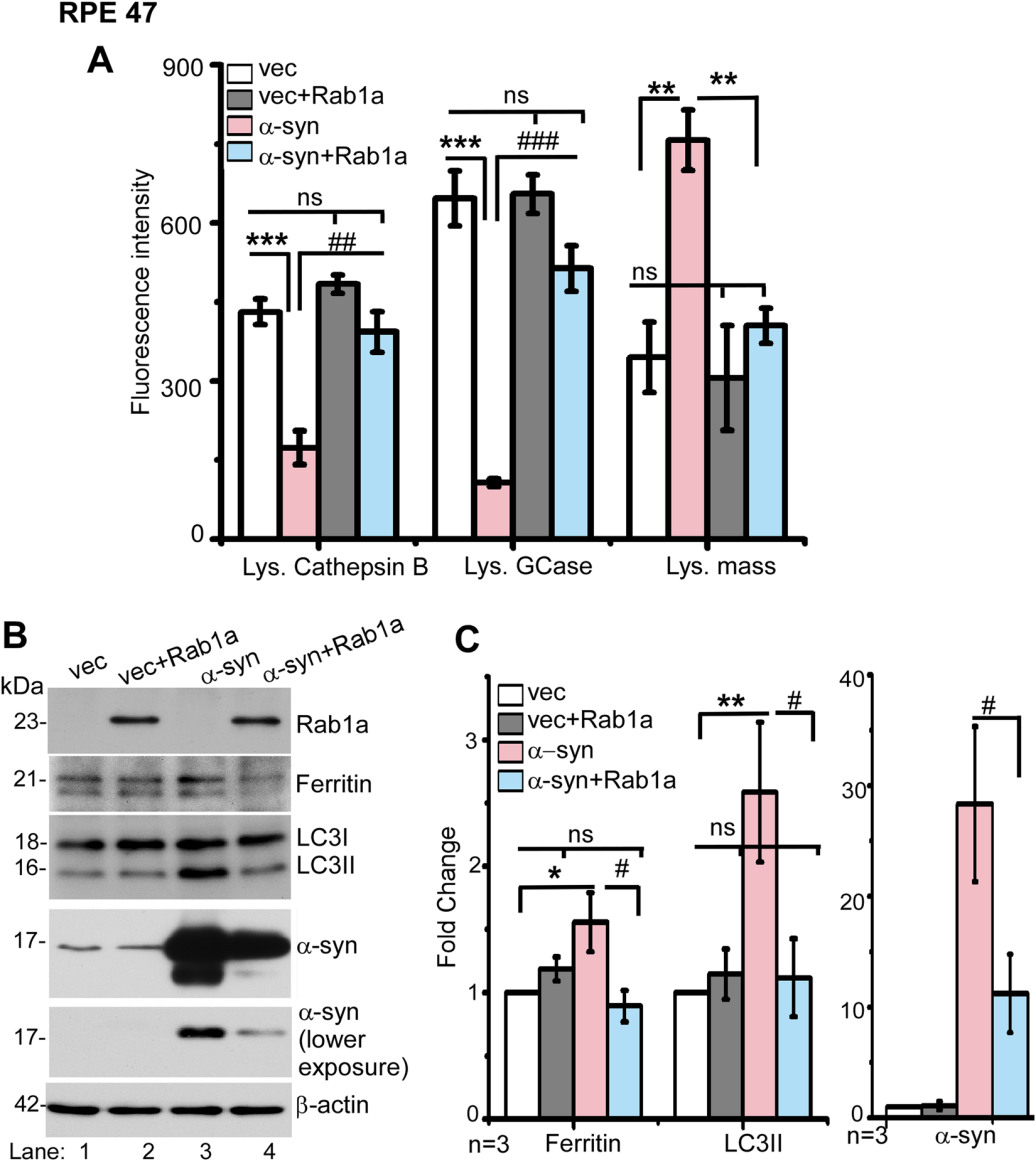
α-Syn-induced lysosomal dysfunction is rescued by Rab1a: (**A**) Quantitative comparison of fluorescence intensity of Cathepsin-B, GCase, and lysosomal mass in vector and α-syn over-expressing cells co-transfected with Rab1a or vector. (**B**) Western blot image of Rab1a, ferritin, LC3, α-syn, and β-actin from vector and α-syn over-expressing cells co-transfected with Rab1a or vector. (**C**) Quantification of ferritin, LC3, and α-syn expression from panel B. All values were normalized to β-actin that served as an internal control. n = 3. *p < 0.05, **p < 0.01, ***p < 0.001, #*p* < 0.05, ##*p* < 0.01, ###*p* < 0.001). Values are mean ± SEM of the indicated *n*. Asterisk (*) and hash (#) signs are used to show comparison with control vector (*) and α-syn (#) expressing cells respectively.

To determine whether impaired degradation of ferritin in RPE cells follows a similar molecular pathway, we quantified the activity of lysosomal hydrolases cathepsin B and glucocerebrocidase (GCase), and measured lysosomal mass in α-syn over-expressing cells^10^. The activity of both cathepsin B and GCase was significantly lower in α-syn over-expressing cells relative to vector transfected controls (Fig. 5A). Lysosomal mass, on the other hand, was significantly higher in α-syn over-expressing cells relative to controls (Fig. 5A). Transfection with Rab1a reversed this phenotype, i.e., the activity of lysosomal hydrolases and lysosomal mass were restored to the levels in vector control, indicating complete rescue of the phenotype (Fig. 5A).

To evaluate whether expression of Rab1a in α-syn over-expressing cells normalizes lysosomal function, cells over-expressing α-syn and vector controls were transfected with Rab1a, and lysates were analyzed by immunoblotting (Fig. 5B). Probing for Rab1a revealed similar expression in the two cell lines, allowing a quantitative comparison of its effect on the expression of ferritin and LC3II, the two proteins that are the subject of this study (Fig. 5B, lanes 2 & 4). A direct comparison of ferritin and LC3II expression in vector and α-syn over-expressing cells revealed higher levels of both proteins in the latter as observed in Fig. 1D above (Fig. 5B, lane 1 vs. 3; Fig. 5C). Expression of exogenous Rab1a, however, reduced the expression of both proteins to levels similar to vector controls (Fig. 5B, lane 4 vs. 1 & 2). Notably, Rab1a caused a significant reduction in α-syn expression, indicating efficient degradation in the lysosomes (Fig. 5B, lanes 3 & 4; Fig. 5C) as reported earlier^10^. The expression of a single adaptor protein Rab1a in RPE cells rescued lysosomal function and degradation of ferritin and α-syn. However, we cannot rule out the possibility that change in the expression of α-syn is due to co-transfection with Rab1a since such a procedure may alter the expression of individual overexpressed proteins.

### Ferritin and α-syn are released from cells in exosomes

**Figure 6:**
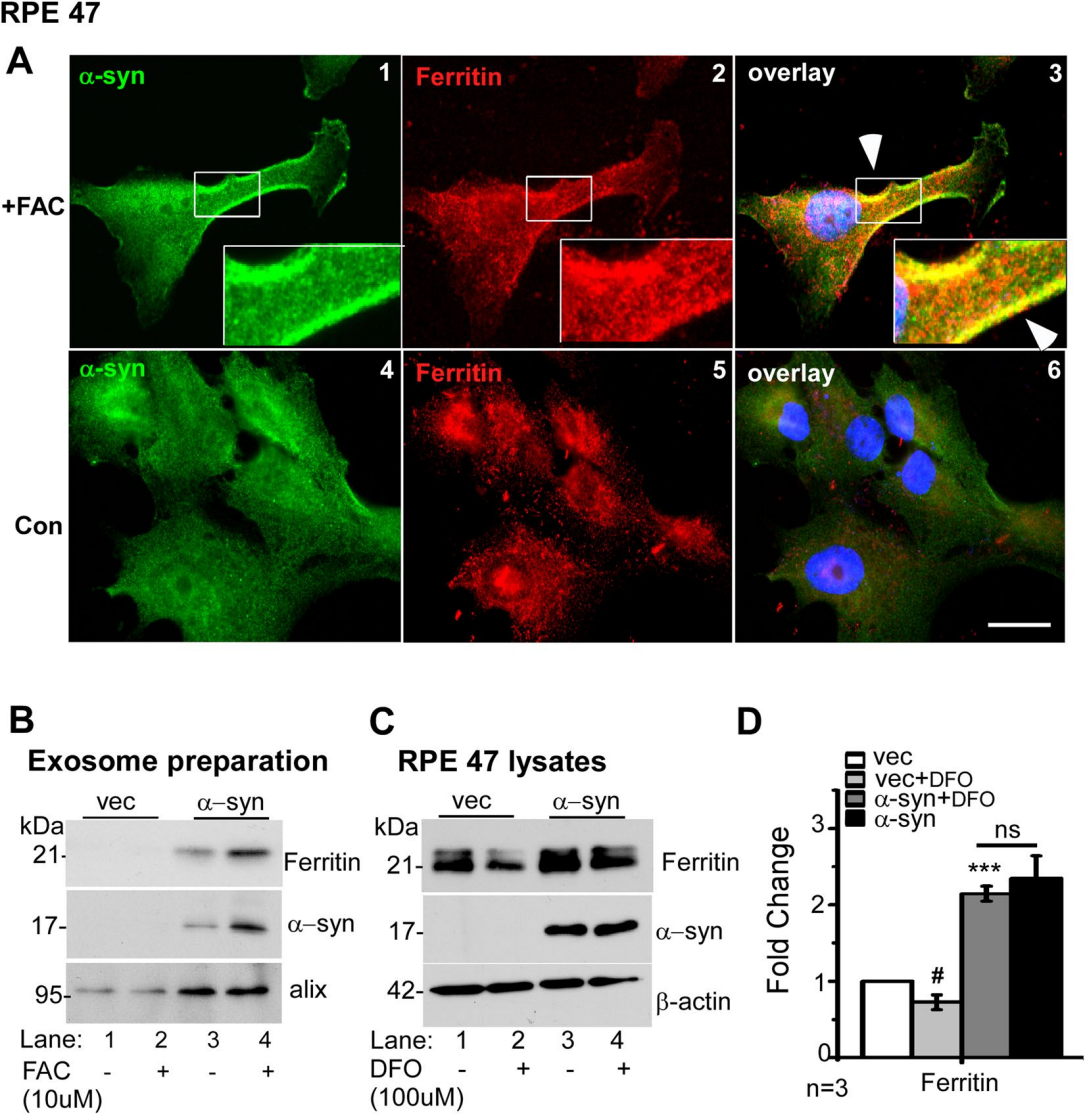
Excess iron induces exosomal release of α-syn and ferritin: (**A**) Co-immunostaining of α-syn-expressing cells exposed to FAC (panels 1–3) or vehicle (panels 4–6) for α-syn (green) and ferritin (red). (**B**) Western blotting image of ferritin, α-syn and the exosomal marker alix from vector and α-syn over-expressing cells exposed to vehicle or FAC. (**C**) Western blot image of ferritin, α-syn, and β-actin from vector and α-syn over-expressing cells exposed DFO. (**D**) Quantification of ferritin from panel C. n = 3 #*p* < 0.05; ***p < 0.001). Asterisk (*) and hash (#) signs are used to show comparison of α-syn over-expressing cells with vector controls (*), and DFO treated vector-expressing cells with untreated controls (#). ns: non-significant.

Exosomes released from RPE cells have been implicated in the pathogenesis of age-related macular degeneration (AMD), the most common cause of blindness in the elderly^41,42^. In addition, soluble and aggregated α-syn are released from cells in exosomal structures, a phenomenon responsible for the prion-like spread of α-syn in PD^43^. To evaluate whether ferritin follows the same path, RPE cells over-expressing α-syn were exposed to an exogenous source of iron (ferric ammonium citrate, FAC) to cause iron-overload, and immunostained. In FAC exposed cells, both α-syn and ferritin were upregulated, and co-localized at the plasma membrane (Fig. 6A, panels 1–3). Control cells cultured in normal medium showed a reaction for both proteins in the cytosol as expected, and no detectable co-localization (Fig. 6A, panels 4–6).

The above results suggested that α-syn and ferritin might be released together in the extracellular medium. To evaluate this possibility, RPE cells over-expressing α-syn and vector controls were exposed to FAC, and exosomes prepared from the culture medium were analyzed by Western blotting. The exosome marker protein Alix was detected in all samples, confirming successful isolation of exosomes by our procedure (Fig. 6B). The recovery of exosomes was significantly more in α-syn expressing cells, and notably, both ferritin and α-syn were present in significant amounts (Fig. 6B, lanes 1 & 2 vs. 3 & 4). Exposure to FAC resulted in significant increase in α-syn and ferritin in the exosomes released from α-syn-expressing cells (Fig. 6B, lane 4 vs. 3).

Thus, α-syn impairs the turnover of ferritin and its own degradation by interfering with lysosomal function, and both proteins are extruded from cells in exosomes.

### α-Syn blocks degradation of ferritin despite cellular iron deficiency

Under conditions of iron deficiency, cytosolic ferritin is degraded in lysosomes to release stored iron^19^. To understand the role of α-syn during iron deficiency, cells were exposed to the iron chelator DFO for 18 h, and lysates from vector and α-syn overexpressing cells were processed for Western blotting and probed for ferritin. As noted above, levels of ferritin were significantly higher in α-syn over-expressing cells relative to vector controls (Fig. 6C lanes 1 vs. 3, Fig. 6D). Exposure to DFO resulted in significant reduction of ferritin in vector expressing cells, but had minimal effect on α-syn overexpressing cells (Fig. 6C lanes 1 vs. 2 & 3 vs 4; Fig. 6D).

## Discussion

We report that overexpression of α-syn in RPE cells impairs degradation of ferritin and release of iron, a process termed ferritinophagy^20^, by compromising lysosomal function. There was a direct correlation between ferritin and α-syn. Downregulation of α-syn decreased ferritin, while overexpression resulted in its accumulation. Both ferritin and α-syn accumulated in LC3 and LAMP1 positive vesicles in α-syn overexpressing cells, which were released in exosomes to the extracellular milieu. α-Syn did not alter the expression of NCOA4, a ferritin chaperone, nor did it interfere with the fusion of autophagosomes with lysosomes. Instead, it disrupted the transport of lysosomal hydrolases to their final destination, a defect reversed by overexpression of Rab1a. These observations partly explain the cause of retinal degeneration in PD, and highlight the role of α-syn in cellular iron homeostasis by modulating the ALP pathway.

The autophagosome/lysosome pathway is of prime importance in RPE cells that are responsible for the turnover of photoreceptor outer segments, a task crucial for retinal function. Several observations from our data indicate that α-syn modulates this activity. There was a direct correlation between α-syn and levels of ferritin and LC3II in the neuroretina of wild-type and α-syn^−/−^ mice, and in RPE cells where α-syn was either silenced or over-expressed. The half-life of ^59^Fe-labeled ferritin almost doubled in α-syn over-expressing cells, suggesting sequestration of iron-loaded ferritin in lysosomes. This is likely to induce a phenotype of functional iron deficiency, partly explaining the upregulation of transferrin receptor (TfR), an iron uptake protein, in α-syn over-expressing cells^30,44^, further increasing the cellular iron load. Modulation of TfR expression by α-syn through Rab1a^45^ and a correlation between TfR expression, ferritin, and LC3^46^ suggests regulation of cellular iron by additional pathways that require further exploration to gain a complete understanding.

Interestingly, α-syn, while known to modulate early steps in the autophagy pathway^47^, is itself degraded by the ALP^48^. Over-expression of α-syn is therefore likely to promote its own accumulation, creating a vicious cycle. Our data indicating decreased activity of cathepsin B and GCase and a significant increase in lysosomal mass in cells over-expressing α-syn indicate that the primary deficiency is in the lysosomal turnover of ferritin and α-syn. Minimal change in the expression of NCOA4 makes it less likely that α-syn interferes with the delivery of ferritin to the phagophore^20,21^. Restoration of ferritinophagy by over-expressing Rab1a confirmed that α-syn disrupts the delivery of lysosomal hydrolases to their correct destination^10^, thus compromising lysosomal function. It is important to note that ferritin was not degraded despite exposure of α-syn overexpressing to DFO, suggesting that iron chelation may not succeed as a therapeutic strategy for PD^49,50^ associated with duplication or triplication of the α-syn gene.

An untoward consequence of lysosomal dysfunction was the release of α-syn to the extracellular milieu in exosomes, supporting the prion-like spread of α-syn aggregates to neighboring cells^43,51,52^. Our observation indicating the release of ferritin along with α-syn is intriguing, and raises important questions. One that is of most concern to PD pathogenesis is whether α-syn and ferritin interact^53^, and the nature of this interaction. Since ferritin released in exosomes is likely to be iron loaded, this interaction could result in the aggregation of α-syn, and prion-like spread of aggregated α-syn and ferritin to adjacent cells.

**Figure 7:**
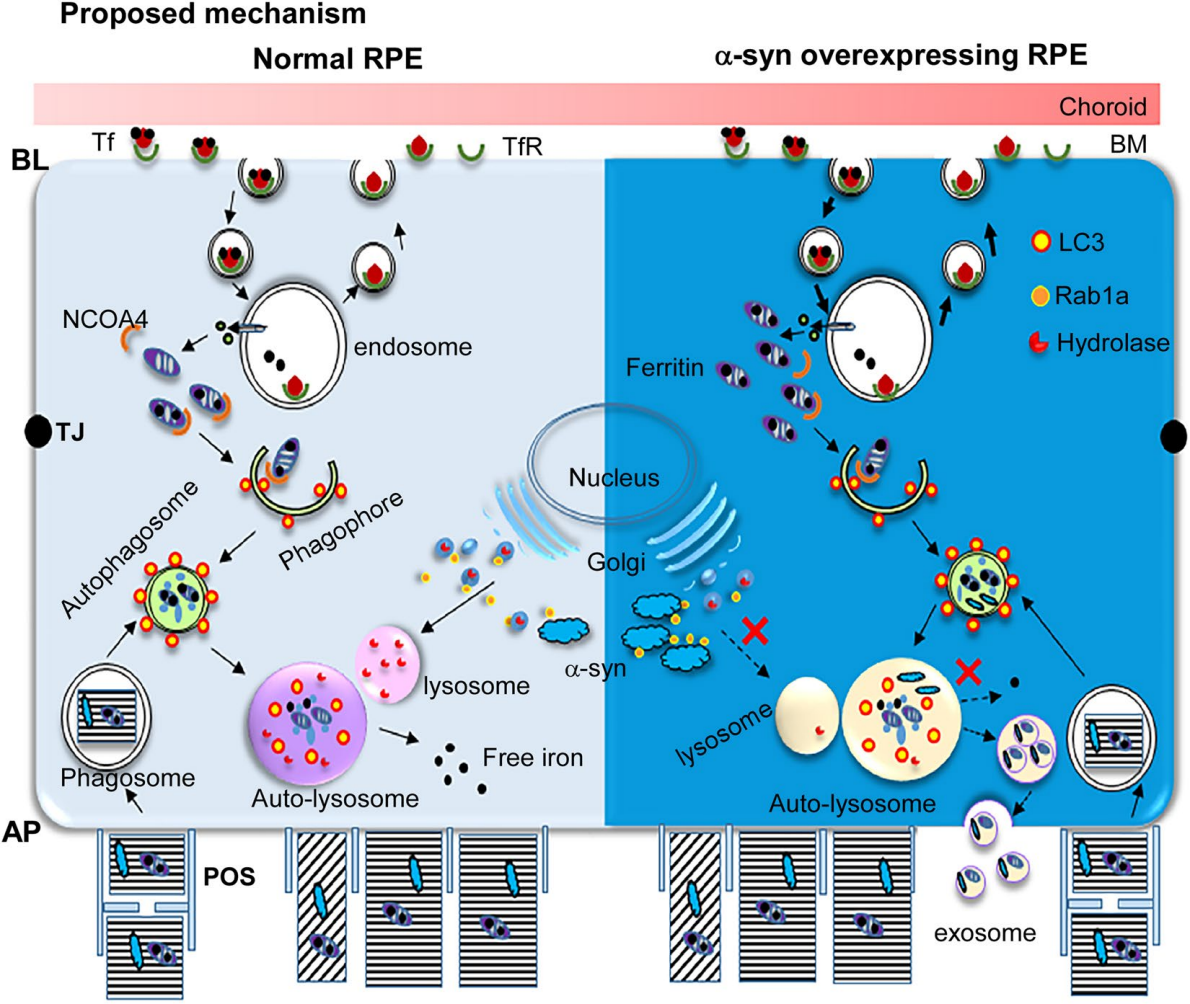
Proposed mechanism. Left panel, wild type RPE cells (light blue): Iron is taken up from the basolateral domain of RPE cells by the classical Tf/TfR pathway, and excess is stored in ferritin. In addition, RPE cells accumulate iron from phagocytosed photoreceptor outer segments that is stored in ferritin. The release of iron from ferritin requires its degradation, a process mediated by NCOA4 that chaperones it to the phagophore that eventually matures into an autophagosome. Autophagosomes fuse with lysosomes, where hydrolases degrade ferritin and release the stored iron. These hydrolases are trafficked to lysosomes from the Golgi with the help of adaptor protein Rab1a. α-Syn is known to interact with Rab1a and inhibit its function, thereby disrupting the trafficking of lysosomal hydrolases^10^. Right panel, α-syn overexpressing RPE cells (dark blue): Over-expression of α-syn sequesters Rab1a, resulting in impaired lysosomal activity and accumulation of iron-rich ferritin in lysosomes. Since α-syn is degraded by the same pathway, inhibition of lysosomal function by α-syn combined with upregulation of ferritin due to iron overloading results in the release of ferritin and α-syn containing exosomes to the extracellular milieu, probably from the AP domain of RPE cells. Abbreviations: AP: apical, BL: basolateral, TJ: tight junction, BM: Bruch’s membrane, Tf: transferrin, TfR: transferrin receptor, NCOA4: nuclear receptor coactivator 4, POS: photoreceptor outer segment.

In conclusion, this study reveals negative regulation of ferritin turnover by α-syn due to impaired lysosomal activity, resulting in the accumulation of α-syn and iron-rich ferritin in lysosomes and their release to the extracellular milieu in exosomes (Fig. 7). This is likely to impact iron cycling in RPE cells, partly explaining the cause of PD-associated retinal pathology. Moreover, the presence of ferritin in α-syn-containing exosomes is likely to promote uptake by neighboring cells *via* the ferritin receptor, a possibility with significant clinical implications that needs further exploration.

## Experimental Procedures

### Animal studies and ethics statement

α-Syn knockout mice (cat # 003692, Jackson Labs, USA) were bred with wild-type C57BL/6NJ to generate homogenous wild type (α-syn^+/+^) and knockout (α-syn^−/−^) mice. Up to three generations were used for experiments^30^. All mouse lines were housed in AAALAC-accredited facilities at Case Western Reserve University (CWRU) School of Medicine (SOM) under a 12h day-night cycle and provided *ad libitum* access to food and water. The animal protocol # 2015–0027 was reviewed and approved by the CWRU IACUC committee in accordance with provisions of the Animal Welfare Act and Guide for the Care and Use and of Laboratory Animals as well as the U.S. Government Principles for the Utilization and Care of Vertebrate Animals Used in Testing, Research, and Training. This facility is directed by Dr. Durfee, DVM, Diplomate ACLAM, and animal care is provided by two full-time veterinarians. The CWRU PHS Assurance number A-3145–01 is valid until 04/30/19.

For all experiments, mice were euthanized and blood was collected by cardiac puncture. The remaining blood was flushed out with 30–50 ml of phosphate buffer saline (137 mM NaCl, 2.7 mM KCl, 10 mM Na_2_HPO_4_, 1.8 mM KH_2_PO_4_). The eyes were enucleated, dissected under a microscope (Leica S4E), and retinal lysates were collected for further experimentation.

### Antibodies and chemicals

The following primary antibodies were used for this study: Ferritin-H (sc-25617) (lot #J2610 and lot #K0713) from Santa Cruz Biotechnology Inc, USA, α-syn from BD transduction, USA (#610786), β-actin (MAB1501) and RPE65 (MAB5428) from Millipore, USA, LAMP1 (ab24170) and NCOA4 (ab56356) from Abcam, USA, LC3B (#2775), Rab1A (#13075) from Cell Signaling Technology, USA. HRP-conjugated secondary antibodies were purchased from GE healthcare, (NA 931 V, NA 934 V) UK, Alexa 488 and Alexa fluor 546 (A11071, A11018) tagged secondary antibodies used for immunostaining were from Molecular Probes, Invitrogen, USA. cathepsin B assay kit was from immunochemistry, USA and PFD-FDGlu and dextran blue were from Thermo Fisher, USA. Ferric ammonium citrate (FAC) (F5879) and DFO (D9533), bafilomycin A1 (B1793), and other chemicals were from Sigma Aldrich, USA.

### Plasmids

Plasmid expressing α-syn has been described before^30^. Briefly the human α-syn cDNA encoding plasmid was purchased from Origene, USA (SC119919) and subcloned in piggybac vector (Systems Biosciences, CA, USA). Rab1a (#46776) and dual tagged LC3-GFP-mCherry (#22418) plasmids were procured from Addgene, MA, USA.

### Cell Culture, transfection and cell treatments

The immortalized human retinal pigment epithelial cell line RPE47 was a kind gift of Dr. Feng Lin, Cleveland Clinic Foundation, OH, USA^54^. Cells were cultured in DMEM (Hyclone, GE, USA) supplemented with 10% heat inactivated FBS (Gibco, Life Technologies, USA) at 37 °C and 5% CO_2_ in a humidified atmosphere, and passaged every third day. Plasmid transfection was performed using lipofectamine 3000 (Invitrogen, USA) according to the manufacturer’s protocol and transfected cells were selected using blasticidin S (Gibco, Life Technologies, USA). Stable cell lines expressing vector or α-syn were used for the studies.

α-Syn siRNA (sc-29619) and control siRNA (sc-37007) were procured from Santa Cruz Biotechnology Inc., USA. RPE47 cells were transfected with siRNA using Lipofectamine RNAi max (Invitrogen, USA) and cultured for 72 h before analysis. Knock-down of α-syn was confirmed by immunoblotting.

To create iron overload or iron deficiency, RPE cells were cultured in the presence of 10 µM ferric ammonium chloride (FAC) or 100 μM deferoxamine (DFO)^19^ respectively in complete DMEM for 18 h at 37 °C. Control cells received an equal volume of vehicle (PBS). Subsequently, the cells were rinsed in PBS and immunostained with specific antibodies or processed for Western blotting.

Bafilomycin A1 is a well-known inhibitor of lysosomal activity, and was used at a concentration of 100 µM for vector and α-syn overexpressing RPE47 cells for 12 h at 37 °C. Control cells were treated with equal volume of DMSO (vehicle). The final concentration of DMSO in the culture medium was 0.625 µl/ml (v/v). Control and treated cells were evaluated by immunostaining or Western blotting.

### Western blotting

Lysates prepared in RIPA lysis buffer (50 mM Tris-Cl pH7.4, 100 mM NaCl, 1% NP-40, 0.5% deoxycholate) were clarified by centrifugation, boiled in reducing gel-loading buffer (50 mM Tris-Cl pH 6.8; 2% (w/v) SDS; 10% (v/v) Glycerol,100 mM β-mercaptoethanol, 0.1% (w/v) bromophenol blue), and fractionated by 15% SDS-PAGE. Proteins were transferred to a PVDF membrane and probed with specific antibodies essentially as described^30^. Dilutions of antibodies were Ferritin-H (1:1000), α-syn (1:2000), β-actin (1:10,000), RPE65 (1:1000), LC3 (1:1000), NCOA4 (1:1000), Rab1a (1:1000), HRP-mouse (1:15,000), and HRP-rabbit (1:15,000). Since ferritin-H antibody (Santacruz-25617) was from two different lots, blots probed with lot # K0713 showed cross-reactivity with ferritin-L chain. For uniformity of quantification we quantified the band corresponding to ferritin-H chain for all experiments.

### Radiolabeling with ^59^Fe

Equal number of vector and α-syn overexpressing cells cultured in 5 ml of DMEM supplemented with 2% FBS were exposed to equal counts of ^59^Fe-citrate for 4 h. The cells were washed with complete DMEM containing 10% FBS and cultured in the same medium for additional 24 h. Subsequently, cells were lysed in native lysis buffer (140 mM NaCl, 1.5% Triton-X-100, 100 mM HEPES pH7.4, 1 mM PMSF) and fractionated on 3–9% gradient native gel^28^. The gel was vacuum-dried and exposed to X-ray film for autoradiography. ^59^Fe-Ferritin band was quantified by densitometry. To control for protein loading, 10 µl of each sample was supplemented with reducing buffer, fractionated by SDS-PAGE, subjected to Western blotting, and probed for β-actin. The identity of ^59^Fe-Ferritin band in the autoradiograph (Fig. 1G) has been confirmed in previous reports where proteins extracted from this band were subjected to Western blotting followed by probing with antibody specific to ferritin, and by transferring the proteins under native conditions and probing transferred proteins for ferritin^33–35^.

### Immunostaining

Cells were cultured overnight on coverslips and fixed in 4% paraformaldehyde for 30 min at room temperature (Sigma Aldrich, USA). Where indicated, cells were permeabilized with 0.1% Triton X-100 for 2–4 min and blocked in 1% BSA before proceeding with immunostaining with the desired primary and secondary antibodies as described^30^. The nuclei were stained with Hoechst (# 33342, Invitrogen, USA), and coverslips were mounted in Fluoromount-G (Southern Biotech, USA) for imaging. Antibody concentrations were as follows: α-syn (1:200), ferritin (1:200), LC3 (1:200), Lamp1 (1:200), alexa-546 & alexa-488 secondary antibodies (mouse & rabbit) (1:1000).

Fluorescent images were captured using a Leica inverted microscope (DMi8) under 63X oil immersion objective. All experiments were repeated a minimum three times. For each experiment images were captured from 20 different fields, and 10–100 cells in each field were evaluated depending on the magnification. To remove bias, all immunostained slides were examined by a second individual blind to the cell line. Representative images are shown in the data

### Dual LC3 Assay

Dual tagged LC3-GFP-mCherry was used to monitor autophagy flux as described diagrammatically in Fig. 2, and explained in the legend. The underlying principle is that GFP fluoresces at neutral pH but is quenched at low pH of the lysosome, while mCherry fluoresces at both neutral and low pH. In short, LC3I is cytosolic, and following lipidation by phosphatidylethanolamine, LC3II associates with autophagosomes^37^. The dual tagged LC3 emits a yellow fluorescence in autophagosomes (green + red). However, when autophagosomes merge with lysosomes, GFP (green) is quenched, while mCherry (red) continues to fluoresce. This method allows estimation of the autophagy-lysosomal flux in a cell.

For the current experiments, dual tagged LC3-GFP-mCherry was transfected in vector and α-syn overexpressing RPE47 cells. The cells were fixed 48 h post transfection and imaged. Green vs. green/red (yellow) vesicles were quantified by analyzing 20 different 40x microscopic fields by two individuals, one blind to the experimental design.

### Lysosomal activity assay

Lysosomal activity was measured as described by Mazzulli *et al*.^10^. In short, RPE cells cultured in 96-well plates were incubated with fluorescent hydrolase substrates for cathepsin B (kit from Immunochemistry, USA) and GCase (5- (pentafluoro-benzoylamino) fluorescein di-β-D-glucopyranoside (PFB-FDGluc) (Life Technologies, USA) for 30 min. Cells were washed 5 times, and substrate degradation was measured in a microplate reader (Synergy 4, Biotek, USA) over 3 h. Activity in the lysosomal compartment was quantified by measuring the response to 100 µM of Baf A1, and normalized to lysosomal mass. Lysosomal mass was measured by cascade dextran blue fluorescence (10 kDa) (1 mg/ml for 6 h) (#D-1976, Life technologies, USA) as described^55,56^.

### Replicates of data and Statistical analysis

All quantification was performed from data obtained from at least three independent (n = 3) experiments. Representative images are shown in the figures. For all images, experiments were repeated a minimum of three times (n = 3). For each experiment, a minimum of 20 random fields were evaluated. Representative images are shown in the figures. Quantification of protein bands was performed by densitometry using UN-SCAN-IT gels (version6.1) software (Silk Scientific) and represented graphically using GraphPad Prism (Version 5.0) software (GraphPad Software Inc). Statistical analysis was done to compare the means of two experimental groups using Graphpad student’s t-test calculator. Asterisk (*) or hash (#) signs were used to indicate statistically significant difference between different groups of samples.

## Electronic supplementary material

Supplementary file
