# Supplementary material for: α-Synuclein impairs ferritinophagy in the retinal pigment epithelium: Implications for retinal iron dyshomeostasis in Parkinson’s disease
Source: Sci Rep. 2017 Oct 9;7:12843. doi: 10.1038/s41598-017-12862-x (PMC5634503; doi:10.1038/s41598-017-12862-x)
Supplement: Supplementary file 1 — Supplementary file [file 41598_2017_12862_MOESM1_ESM.pdf]

α-Synuclein impairs ferritinophagy in the retinal pigment epithelium: Implications for retinal iron dyshomeostasis in Parkinson's disease

Shounak Baksi, Neena Singh*

***Corresponding author**
E-mail: neena.singh@case.edu

Department of Pathology, School of Medicine, Case Western Reserve University, Cleveland, Ohio 44106, USA.

Full gel images of indicated Figure numbers

Figure 1A

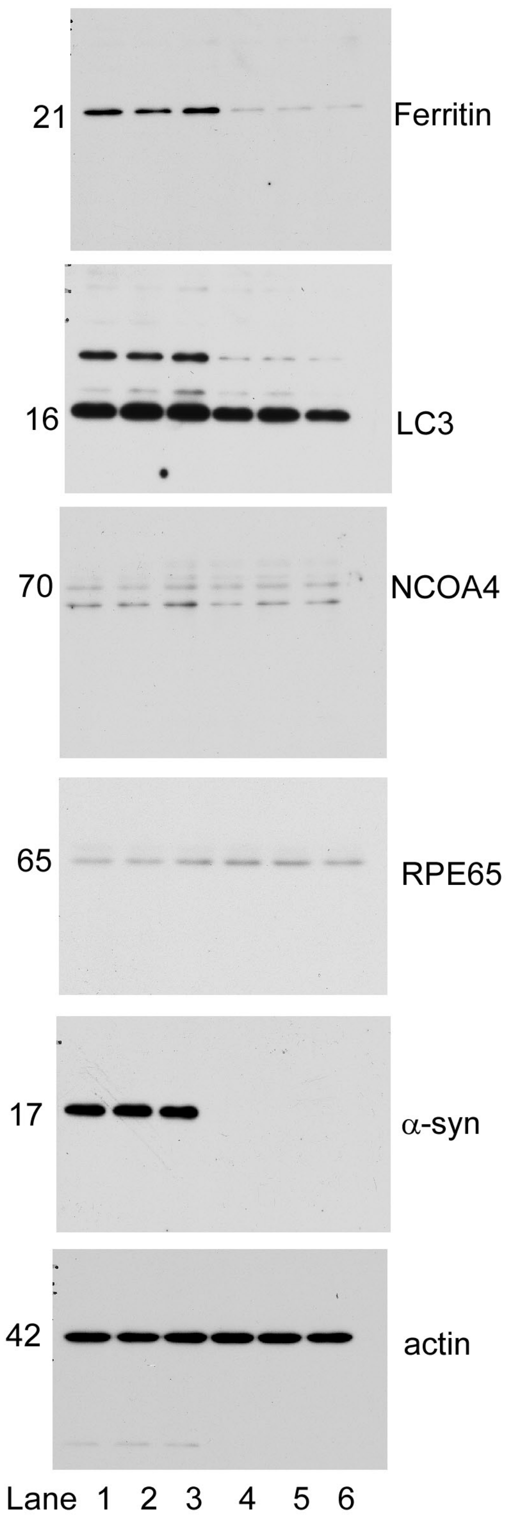

samples: Lanes 1-3 : α-syn+/+
lanes 4-6:α-syn-/-

Figure 1C

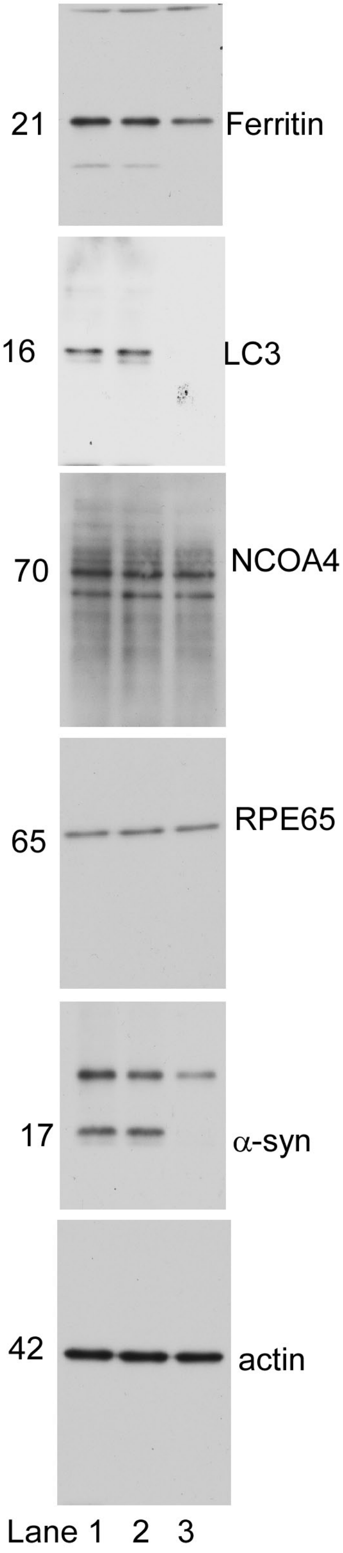

samples
1. non transfected
2. vector
3. α-syn transfected

Figure 1D

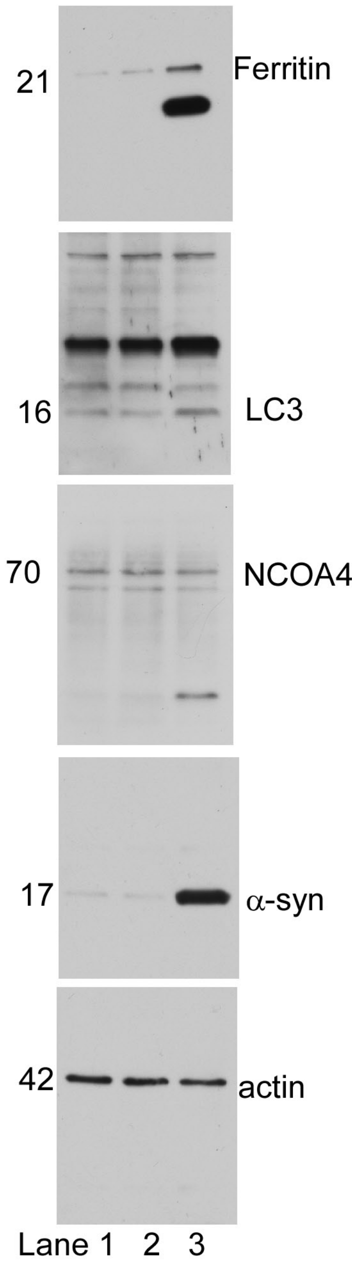

samples
1. non transfected
2. con siRNA
3. α-syn siRNA

Figure 1G

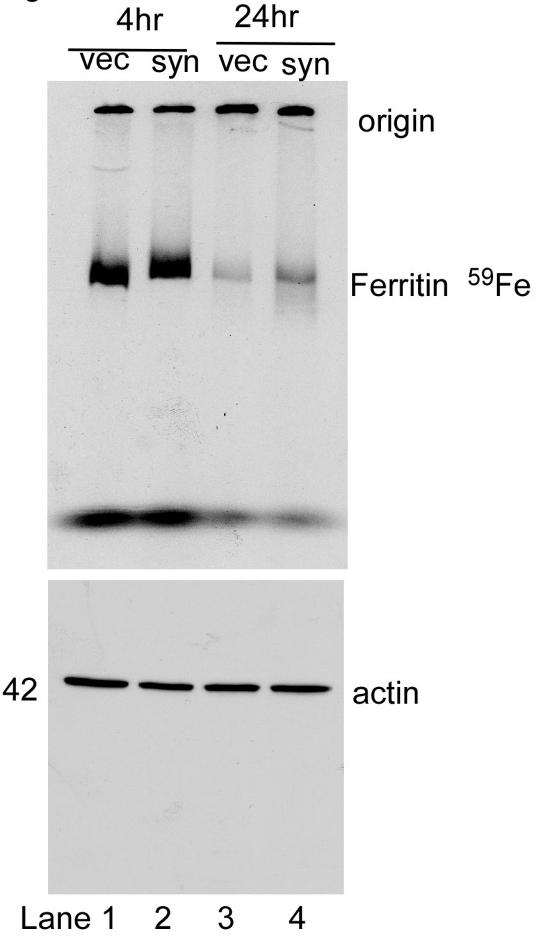

Figure 2C

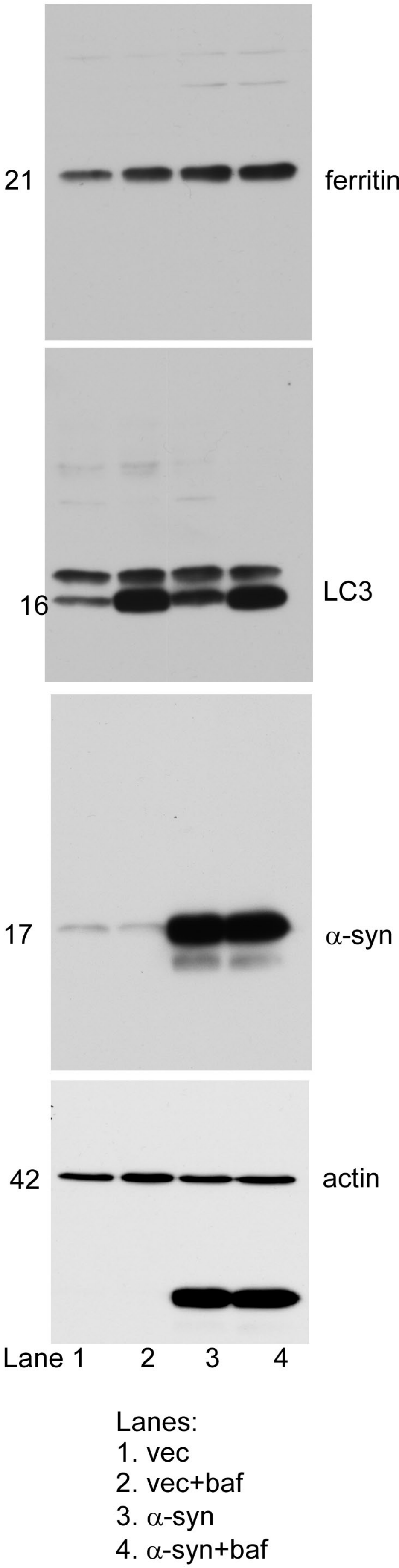

Figure 4A

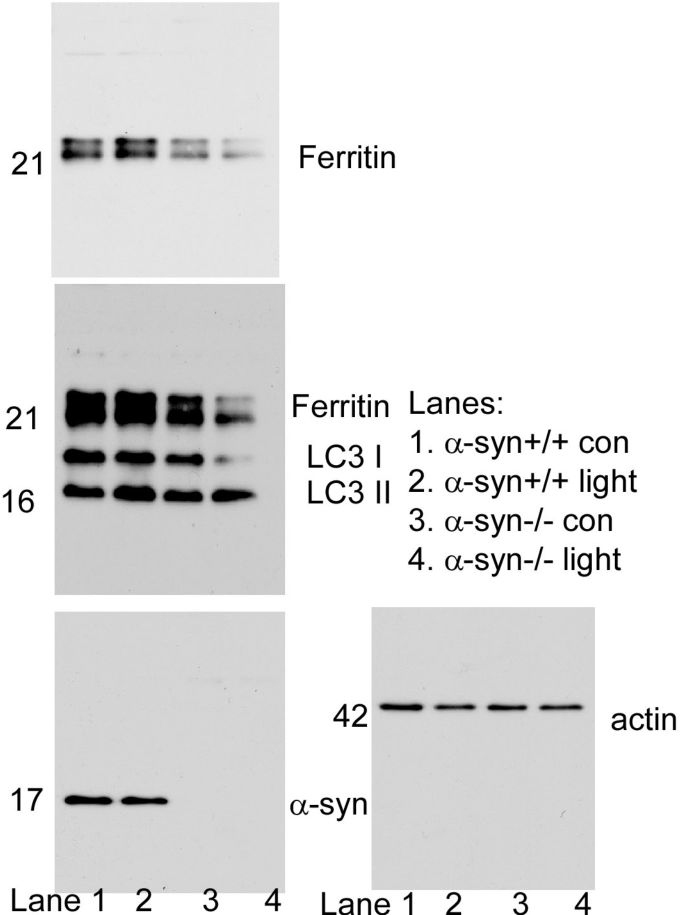

Figure 5 B

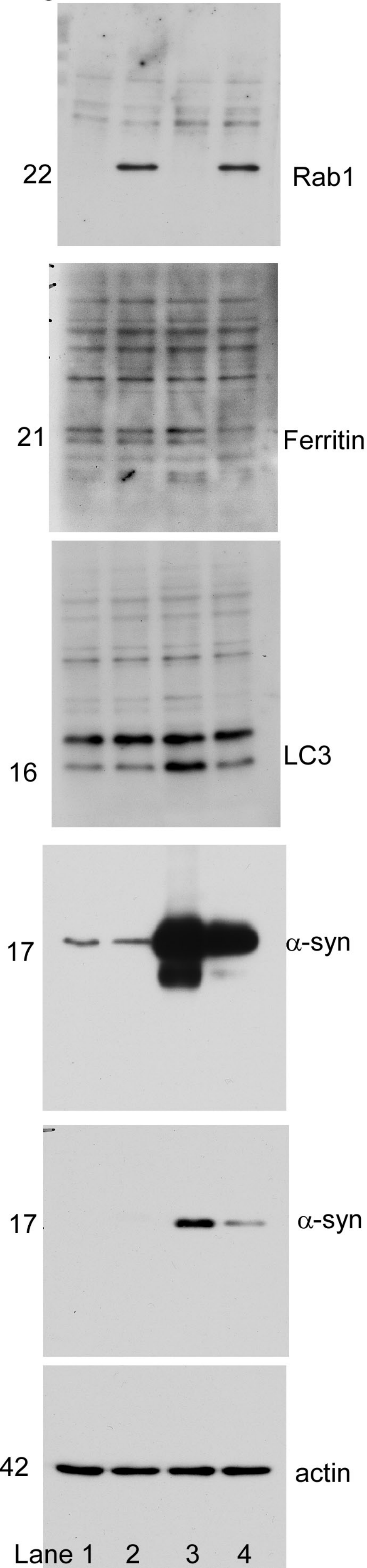

samples:
1. vec
2.vec+Rab1
3. α-syn
4. α-syn+Rab1

Figure 6 B

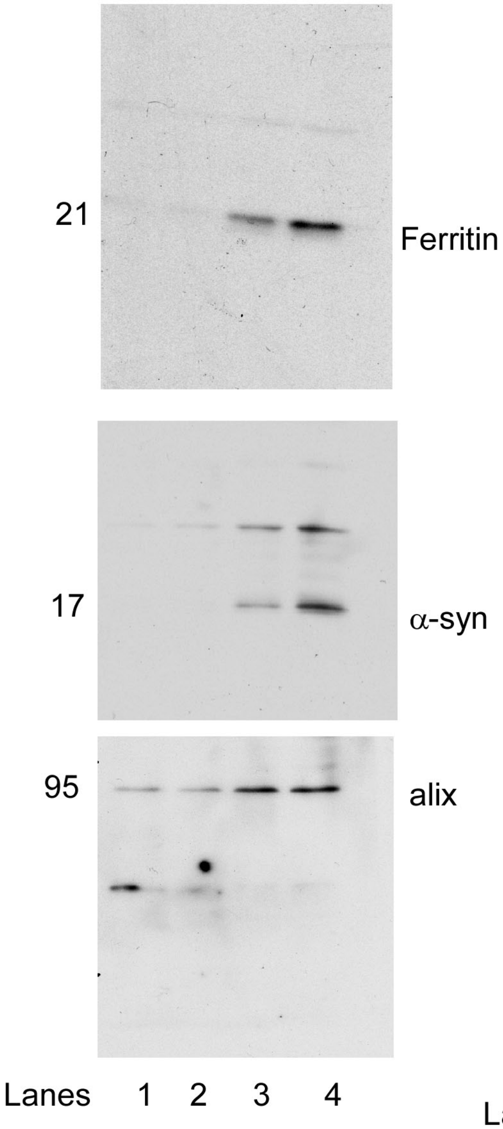

samples:
1. vec
2.vec+FAC
3. α-syn
4. α-syn+FAC

Figure 6 C

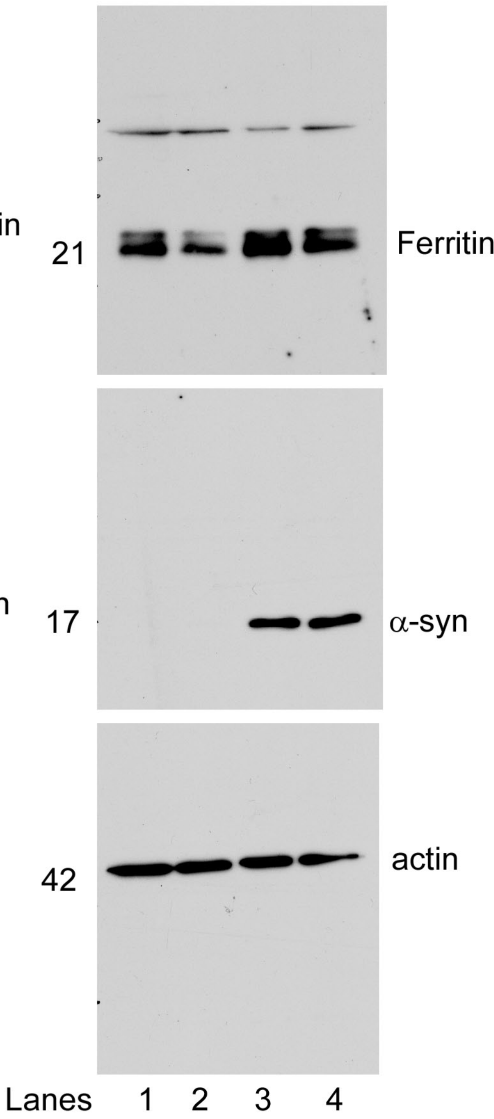

samples:
1. vec
2.vec+DFO
3. α-syn
4. α-syn+DFO

Supplementary Figure legends

Full gel images for

Figure 1 A: All images are exposures from same gel. Sequence of antibody probing Ferritin, LC3, RPE65, NCOA4, α-synuclein, β-actin.

Figure 1C: All images are exposures from same gel. Sequence of antibody probing- LC3, ferritin, RPE65, NCOA4, α-synuclein, β-actin.

Figure 1D: All images are exposures from same gel. Sequence of antibody probing- α-synuclein, ferritin, LC3, NCOA4, β-actin.

Figure 1G: Images shown are from diferent gels. 59-Fe autoradiography from a native gel with indicated sample loads. β-actin image from equal amount of samples run on a SDS-PAGE and western blotted.

Figure 2C: All images are exposures from same gel. Sequence of antibody probing- ferritin, LC3, α-synuclein, β-actin.

Figure 4A: All images are exposures from same gel. Sequence of antibody probing- ferritin, LC3, α-synuclein, β-actin.

Figure 5B: All images are exposures from same gel. Sequence of antibody probing- Rab1a, ferritin, LC3, α-synuclein, β-actin.

Figure 6B: All images are exposures from same gel. Sequence of antibody probing- ferritin, LC3, Alix.

Figure 6C: All images are exposures from same gel. Sequence of antibody probing- ferritin, α-synuclein, β-actin
